# Impairment of the serotonergic neurons underlying reinforcement elicits extinction of the repeatedly reactivated context memory

**DOI:** 10.1038/srep36933

**Published:** 2016-11-14

**Authors:** Pavel M. Balaban, Alia Kh. Vinarskaya, Alena B. Zuzina, Victor N. Ierusalimsky, Aleksey Yu. Malyshev

**Affiliations:** 1 Institute of Higher Nervous Activity and Neurophysiology, Russian Academy of Sciences, Moscow, Russia; 2 Lomonosov Moscow State University, Moscow, Russia

## Abstract

We analyzed changes in the activity of individually identifiable neurons involved in the networks underlying feeding and withdrawal behaviors in snails before, during, and after aversive learning *in vitro*. Responses to food in the “reinforcing” serotonergic neurons involved in withdrawal changed significantly after training, implying that these serotonergic cells participate in the reactivation of memory and are involved in the reconsolidation process. In behavioral experiments it was shown that impairment of the functioning of the serotonergic system with the selective neurotoxin 5,7-DiHT did not change the memory, when tested once, but resulted in a complete extinction of the contextual memory after repeated reactivation of memory. Conversely, the cued memory to a specific type of food was significantly reduced but still present. Thus, we conclude that it is only for the context memory, that participation of the “reinforcing” serotonergic neurons in memory retrieval may be the gate condition for the choice between extinction/reconsolidation.

One of the most interesting properties of memory is that a stable consolidated memory can be disturbed by the same factors (*e.g.* protein synthesis blockade) that can impair newly formed memories shortly after acquisition, if applied along with the “reminder” cues representing a part of the learning situation^1^,^2^,^3^,^4^. The concept of reconsolidation assumes that newly acquired memories are not consolidated once and remain intact forever^5^. Several reports have shown that after the presentation of a specific reminder, reactivated old memories become labile and again susceptible to amnesic agents. This vulnerability diminishes with the progress of time and implies a re-stabilization phase, usually referred to as reconsolidation^3^,^6^.

Context-specific learning and memory have been shown in many invertebrates^7^,^8^. In terrestrial snails contextual memory was described in detail^9^,^10^,^11^, by demonstrating^12^ that the protein synthesis blocker anisomycin impairs contextual memory if injected immediately after a “reminding” session, which suggests the existence of a reconsolidation phase after memory reactivation. Reactivation of a consolidated memory resulting in the reconsolidation phenomenon has also been shown in other invertebrates^13^,^14^,^15^,^16^,^17^,^18^. Recently, a reconsolidation-like process has been demonstrated in neural networks consisting of co-cultured Aplysia neurons at the level of single sensory-to-motor neuron synapses^19^,^20^.

There is a growing body of experimental data implicating serotonin (5-hydroxytryptamine, 5 -HT) in a wide range of memory processes in molluscs^21^,^22^, as well as in vertebrates^23^,^24^. The evidence for a specific role of 5-HT in the induction of long-term sensitization^25^, and in the cellular mechanisms of classical conditioning^26^,^27^,^28^ suggests possible roles for 5-HT in the acquisition of memory. However, participation of 5-HT in the reconsolidation phenomenon and in retrieval of memory has not been investigated recently.

One of the approaches that may be employed for the investigation into the role of 5-HT in behavioral plasticity is selective impairment of the serotonergic neurons with neurotoxins. Both 5,6-DiHT and 5,7-DiHT (5,7-dihydroxytryptamine) are known to be sequestered selectively within serotonergic neurons by a high affinity uptake system^29^. The neurotoxin is oxidized intracellularly, producing free radicals which elicit a significant decrease in 5-HT production starting from the 3^rd^ day^30^, and ablation of serotonergic terminals both in vertebrates^31^ and invertebrates^30^,^32^. Using this approach, a specific role of individually identifiable serotonergic neurons was shown in the cellular and behavioral plasticity of the terrestrial pulmonate snail *Helix lucorum*^11^,^27^,^33^,^34^. Intracellular activation of a single identified serotonergic cell was shown to be sufficient to elicit the associative changes in monosynaptic connections via an increase of [Ca^2+^] in the postsynaptic cell in a simple network consisting of 3 neurons which have defined behavioral roles in the withdrawal behavior of *Helix*^33^,^34^.

In the present paper the contribution of 5-HT to reconsolidation was studied in terrestrial snails using electrophysiological experiments with intracellular recording. This method was chosen for its advantage of being able to record intracellularly from functionally and morphologically identified serotonergic and other neurons before and after associative training *in vitro*. Behavioral experiments using 5,7-DiHT were also used. In the behavioral experiments, the associative changes were compared after training in normal and 5,7-DiHT-injected snails, using a paradigm for testing the existence of reconsolidation (retrieval of memory without reinforcement under blockade of protein synthesis) of the contextual and cued aversion memories. The results suggest that extinction of memory (forgetting) may be the result of a lack of activity in the reinforcing neurons during repeated retrieval of memory.

## Methods

Experiments were carried out in adult *Helix lucorum taurica L.* specimens weighing 20–22 g. The snails were kept in a wet environment and fed as usual with carrots. Three to 5 days before training sessions the experimental animals were deprived of food. The experimental procedures were in compliance with the Guide for the Care and Use of Laboratory Animals published by the National Institute of Health, and the protocol was approved by the Ethical Committee of the Institute of Higher Nervous Activity and Neurophysiology RAS.

### Electrophysiological experiments

**Figure 1:**
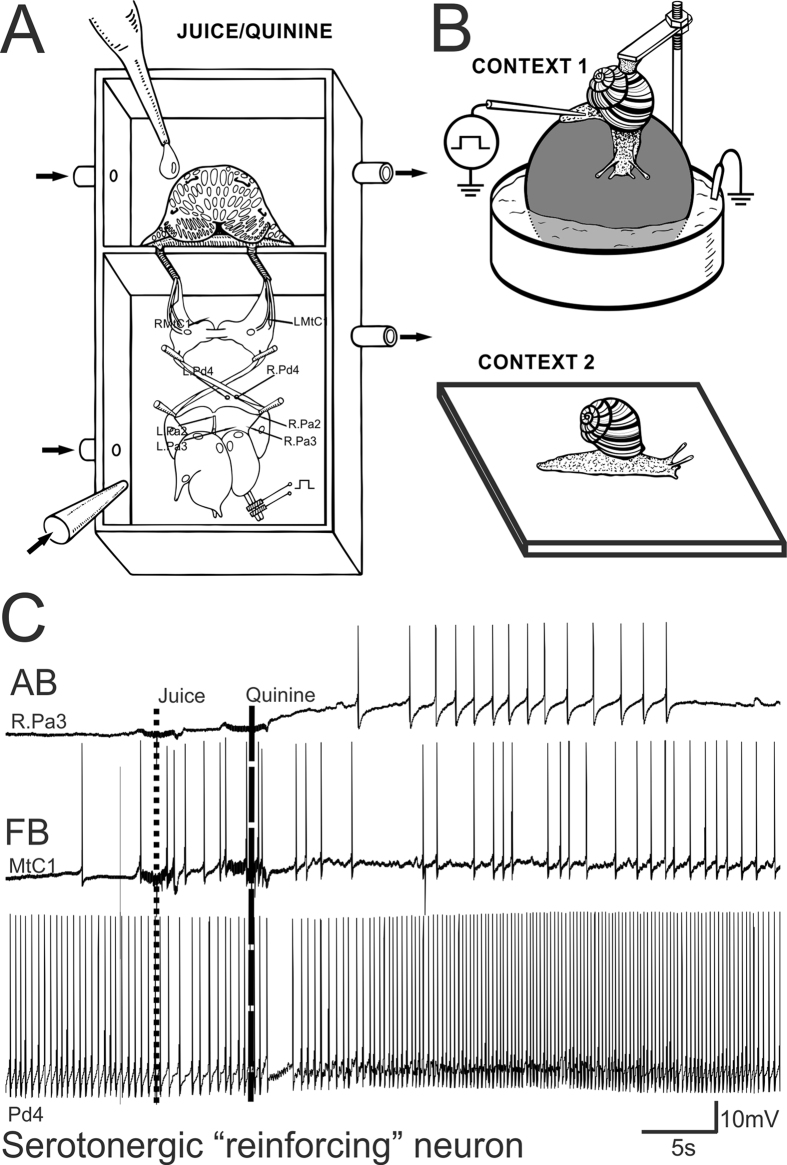
(**A**) Schematic representation of the two-compartment experimental chamber with the preparation. The compartment containing the CNS was continuously perfused with the snail physiological solution. After application of a drop of carrot juice or quinine to the lip it was washed out with the same solution. The neurons, the activity of which was recorded in neurophysiological experiments were: RMtCI. LMtCI, right and left giant meta- cerebral neurons; RPd4, LPd4, right and left pedal serotonergic neurons; RPa2, RPa3, LPa2, LPa3, giant neurons located in both parietal ganglia. (**B**) Schematic representation of Context 1 and Context 2 in which the snails were trained (shocks in Context 1 only) and tested for tentacle withdrawal amplitude. (**C**) Example of recordings from 3 functionally different neurons during a pairing trial. Upper trace – recording from a giant parietal (RPa3) premotor interneuron involved in triggering the withdrawal; middle trace – recording from a serotonergic cerebral giant neuron (MtC1) involved in feeding; lower trace – a recording from a serotonergic pedal cell (Pd4) modulating synaptic inputs of parietal premotor neurons. Dotted line shows time of juice application on the lip, dashed line shows time of quinine application on the lip. Notice the lack of response to juice in withdrawal interneuron (upper trace) and pedal serotonergic cell (lower trace), while the feeding interneuron responds both to juice and quinine (middle trace). AB, avoidance behavior and FB, feeding behavior.

The neurophysiological experiments were carried out on a ‘CNS-lip’ preparation consisting of the central nervous system and the head region containing the tentacles, mouth and lips. Details of the preparation, and the identification of neurons are given elsewhere^11^. Briefly, animals were cooled to 4 °C and injected with isotonic MgCl_2_, before CNS isolation in order to minimize pain. The central ganglia complex and lip were surgically isolated from anesthetized snails with special attention to the preservation of the cerebral ganglia nerves connecting the cerebral ganlia and head of the animal. The ganglia and head were placed in a two-compartment experimental chamber (Fig. 1A) and the slits in the separation wall through which the nerves passed were filled with Vaseline. All nerves connecting the cerebral ganglia with the head remained intact in the lip-CNS preparations. Tentacles were cut off from the *musculi columellaris* and were preserved in preparation together with the oral part of the head. A drop of carrot juice applied to the surface of the lip served as a conditioned stimulus (CS), and a drop of quinine chloride (10^−3^ mol/l) as a reinforcing (negative) one. The conditioned reflex was elaborated on the ‘CNS-lip’ preparation (Fig. 1A) with the possibility to record changes in responses to the CS before, during and after pairings. The compartment containing the CNS was continuously perfused with snail physiological saline (concentration in mM: 100 Na^+^, 4.2 K^+^, 7.0 Ca^2+^, 4.6 Mg^2+^, 127.4 Cl^−^, 10 HEPES, pH 7.6) at a rate of 2 ml/min. After the application of a drop of carrot juice or quinine to the lip, the chamber was washed out with the same saline. We recorded the activity of the following readily identifiable neurons in neurophysiological experiments: the right and left giant metacerebral neurons (RMtCI, LMtCI) involved in feeding behavior; the giant premotor neurons located in both parietal ganglia involved in triggering withdrawal (RPa2, RPa3, LPa2, LPa3); the left and right pedal serotonergic neurons (LPd4 or RPd4) mediating the reinforcement in the withdrawal network of the terrestrial snail (shown in Fig. 1A, details and map in ref. 11). Trials were given with 10 min intervals. Associative changes were tested at least 90 min after the last reinforcement. The intracellular activity of up to 4 identified neurons (marked on Fig. 1A) was recorded simultaneously using conventional microelectrode techniques. Intracellular signals were recorded with preamplifiers (Axoclamp 2B, Axon Instruments, USA), digitized, and stored on a computer (Digidata 1400 A A/D converter and Axoscope 10.0 software, both from Molecular Devices, USA).

### Behavioral experiments: context conditioning paradigm

In the experimental set-up (Fig. 1B, Context 1), the snail was tethered by its shell in a manner allowing it to crawl on a ball that rotated freely in a water solution containing 0.01% NaCl (Fig. 1B, upper inset). The ball was covered with aluminium foil to complete an electrical circuit between the animal’s foot and a carbon electrode placed in the water. Electric shocks were delivered using a 1 to 4 mA, 1 sec current through a macroelectrode applied manually to the dorsal surface of the snail’s foot. Punctate mechanical stimuli were applied with calibrated von Frey hairs, permitting the delivery of pressures ranging from 6 (estimated as weak) to 68 gr/mm^2^ (estimated as noxious). After several pilot series, the behavioral response, the intensity (25 gr/mm^2^), and the location of tactile stimulation were chosen. The withdrawal amplitude of ommathophores (posterior tentacles) in response to the chosen intensity of tactile stimulation of the rostral part of skin (4–5 mm behind the posterior tentacles) was 10–30% of the maximal level in normal animals. In pilot experiments, it was shown that responses to such test stimulation were sensitized after noxious stimuli, and this part of the foot skin was chosen as the standard place for tactile stimulation. An investigator, blind to the experimental histories of the animals, applied the tactile stimuli to the snail’s skin and recorded the tentacle withdrawal on video. To quantify and average the results, we analyzed the distance between tip and base of the tentacle off-line, and scored the withdrawal amplitude as a percentage of the initial length of the tentacle in each trial.

Before training, each snail was exposed to the experimental set up for 30 min daily for two days. Then the first test session (T0) was performed for all groups. Blind testing was performed for each snail in two alternating contexts (Context 1 was a ball floating in water, while Context 2 was a flat glass similar to the glass of the terrarium where the snails were kept between the experimental sessions, see Fig. 1B). After obtaining the pre-training scores, each group of snails received one or two electrical shocks per day with 20–30 min intervals for 10 days in Context 1 alone. The current magnitude was individually chosen for each snail so that a complete withdrawal of the anterior part of the body was observed in response to a shock. No testing was performed during the training sessions. On the second day after completion of the training session (animals were fed during the rest period in the terrarium), the responsiveness to the same test tactile stimuli (T1,) was compared in all parallel groups of snails. The order in which the animals were tested in each context was randomized. To reduce the possible effects of recent handling, the test was administered no sooner than 5 min after the subjects had been placed in the environment. Only actively moving animals were tested. The neurotoxin 5,7-DiHT was injected 1 hr after the T1 session.

48 hours after the second test session (T1), all groups of snails were reminded of training by placing the snails in the same context where they were previously shocked (on the ball, Fig. 1B) for for 20 min (Reminder). Twenty minutes before the reminder, the snails were injected either with anisomycin, mianserin, or saline (for details of dissolving and concentration calculations see the*“Drugs and injections”* section). On the following days after a session of testing, drug injections and “reminding”, the third, fourth and fifth test sessions (T2-4) were performed for all parallel groups in two different contexts without any drug injections.

### Behavioral experiments: food aversion paradigm

Conditioned food-aversion in snails was shown quite a few years ago (for review see ref. 10), and we used the same paradigm as before. A total of 25 snails were trained in the present series of experiments. Before training the animals were deprived of food for 3 days, and no food was given throughout the experiment. During training sessions, the animals were fixed by their shells to a horizontal holder in such way that they could crawl on a plastic ball floating in water (Fig. 1B, upper context). As a conditioned stimulus, a small cotton swab soaked in fresh carrot juice (carrot is a preferred food for snails) was placed with a mechanical manipulator at a distance of 5 mm from the rhinophores (lower small tentacles) of the snail crawling on the ball. Electric stimulation (to negatively reinforce the feeding response) was delivered using a 1–6 mA, 0.5 s electrical shock with a macroelectrode placed manually on the dorsal surface of the snail’s foot, and the second electrode (a carbon one) was placed in the water, which had a low concentration of NaC1. Every snail was presented food and equal numbers of electric shocks (5 trials in a training day). Electric shocks were delivered to the conditioned animals exactly at the time of the first attempt to contact and bite the source of the odor. In each experiment we chose a moderate intensity of the shock in order to elicit head and tentacle withdrawal accompanied by a weak mucus release, but not whole body withdrawal into the shell. The criterion time for the feeding response was taken as 120 s: in the cases when the snail did not try to bite the “food” during this period, the behavioral response was scored as a refusal of food. The percentage and latencies of feeding responses (when the animal tried to consume food during the criterion time) were scored before and after training (5 presentations of food with 10–20 min intervals). The latency of the first attempt to bite the “food” was scored. The criterion for the elaboration of a conditioned aversion to food was taken as 4 successive refusals. This criterion was usually reached after 4 sessions/days of paired presentations of food and electric shock. Blind testing of associative changes in behavioral responses to food after the training, drug injections, etc. were carried out according to protocol^10^. After 1 day of rest after the training, the next test was performed (T1), and each animal was blind tested 3 times with 5 min intervals. On the same day, after testing, animals from Group 3 were injected with 5,7-DiHT (20 mg/kg) and all snails were rested for two days. Before the next test (T2) all snails received either sham injections (Group 1 and 3), anisomycin (Group 2, 0.4 mg per snail) or mianserin, a nonselective serotonin receptor antagonist (final concentration in body 0.05 mM). All drugs were injected at a volume of 0.5 ml. Thirty min after injections, all animals were reminded by placing them in the experimental context and presenting the reinforced odor 3 times. For five consecutive days all animals were tested 3 times in one 20 min session for maintenance of food-aversion memory (T3-T7) without injections, except Group 4 which was injected with mianserin each day after the test.

### Drugs and injections

Anisomycin (ANI, from SIGMA) was dissolved in sterile saline, adding an equimolar amount of 3 N HCl and adjusting the pH of the resulting solution to 7.2 with 3 N NaOH. The snails were injected with 5,7-DiHT (dihydroxytryptamine creatinine sulphate from Sigma) at a total volume of 0.7 ml, in doses of 20 mg/kg dissolved in sterile Ringer saline (in mM: 100 NaCl, 4 KCl, 7 CaCl2, 5 MgCl2, and 10 Tris-HCl buffer (pH 7.8) containing 1 mg/ml ascorbic acid as antioxidant. Control animals received an injection of the same volume of vehicle. Mianserin (SIGMA) was dissolved in warm deionized water, injected at a calculated final concentration of 5 × 10^–5^ M in the body of the animal.

For calculating final concentrations in the body, each gram of the snail’s body weight was scored as 1 ml. Drugs for *in vivo* injections were prepared in deionized water as a stock solution at a concentration 28.6-fold greater than required. Because the snails used in these experiments were comparable in weight (20 g ± 2), 0.7 ml of the drug solutions were injected into the hemocoel, thereby achieving the required concentration in the animals’ body (0.7 ml × 28.6 = 20 ml).

Intracoelomic injections were performed with a fine needle via an insensitive part of the foot skin normally hidden under the shell. During injections, the snails stopped locomotion and lowered the ommatophores, which was most likely due to the experimenter raising the shell. However, the snails never showed a generalized withdrawal into the shell.

Only animals that survived all treatments and were in good condition for a week after experiments were considered for statistical evaluation. Morphological evidence of serotonergic neuron lesions (brown granular staining of serotonergic neurons 30 and more days after the injection, see ref. 35) was checked after the experiments, and was present in all treated animals. A blind procedure was used throughout the experiments.

### Retrograde labeling via nerves

We performed retrograde labeling of the neurons in the left cerebral and left pedal ganglia via the cerebro-pedal connective. The cut end of the connective was sucked into a pipette filled with 10% Neurobiotin (Vector Labs) in 0.1 M KCl. The end of the nerve was left in place for 12–24 hours at 18–22 °C. The time of backfill was chosen experimentally. The ganglia were fixed with 4% paraformaldehyde in 0.1 M phosphate buffer for 2 hours. The brains were processed with the Vectastain ABC kit (Vector Labs).

#### Statistical evaluation of data

Blind testing was performed in all behavioral experiments. Comparisons between groups were only made for parallel groups of animals in one experimental series. We used the nonparametric Mann-Whitney rank sum test to compare performance of two groups of snails, and the Wilcoxon signed-rank test was used for the comparison of performance of the same group in different conditions. ANOVA was used when more than two groups of animals were compared. Significant differences in performance are indicated on all figures.

## Results

### Electrophysiological experiments

In the present study we decided to use the advantage of a ‘CNS-lip’ preparation (Fig. 1A) that allows recordings to be taken before, during and after the training sessions. With the CNS-lip preparation, the activity of functionally identified neurons belonging to 3 different functional classes can be recorded. We chose to record from the metacerebral giant serotonergic neurons (MtC1, left or right) that are known to participate in feeding behavior (FB – feeding behavior, middle trace on Fig. 1C) and to receive information about all kinds of chemical stimulation in order to monitor the conditioned stimulus (drop of juice) and the reinforcement (drop of quinine) perception in each preparation. In parallel we recorded from one of the 4 giant parietal premotor withdrawal interneurons (AB – avoidance behavior, upper trace on Fig. 1C) known to trigger the withdrawal, to respond to all noxious stimuli and change their responses to food after aversive conditioning^27^.

The neuromodulatory giant serotonergic pedal cells Pd4 are usually tonically active and respond with long-lasting excitation to all noxious stimuli^27^,^36^. Intracellularly evoked activity of these neurons can serve as a reinforcement for elaboration of associative changes in amplitudes of synaptic inputs to parietal giant neurons triggering the withdrawal of tentacles^33^. In each experiment we recorded from the left or right serotonergic pedal #4 neurons in order to monitor changes due to the training.

An example of a trial in which a drop of carrot juice was followed by a drop of quinine is shown in Fig. 1C. No response to juice was observed in a 10 sec interval in a neuron involved in avoidance behavior (upper trace), and in the pedal serotonergic neuron (lower trace). The cerebral neuron involved in feeding behavior (middle trace) responded to juice with several action potentials showing that information about juice is perceived. Application of quinine elicited a strong depolarization in avoidance behavior neuron followed by a spike discharge, moderate response in a feeding behavior neuron, and a significant increase in frequency of spikes in pedal serotonergic neuron (lower trace). The maximum response to the reinforcing stimulus in all neurons was 15–20 sec after the application of a drop of quinine.

The results of all electrophysiological experiments are summarized in Table 1. Statistical evaluation of raw data before and after training in preparations (mV for Pa2/3 and frequency in % for other neurons) showed that changes in responsiveness in serotonergic #4 pedal neurons and premotor withdrawal interneurons #2/3 after training were highly significant (Wilcoxon Signed Rank Test, P < 0,001), while changes in the metacerebral neuron (MtC1) involved in feeding behavior were non-significant.

**Figure 2:**
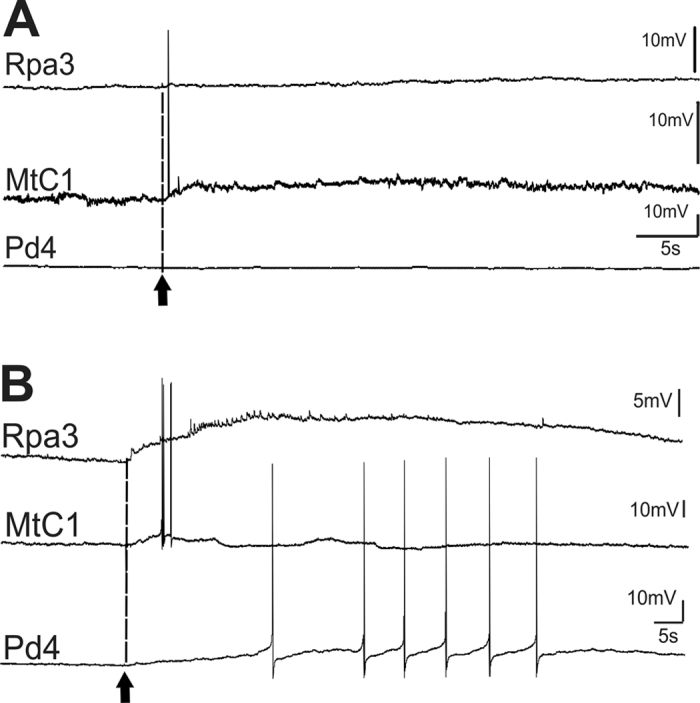
Example of changes of responses to juice application (arrow and dashed line) in neurons functionally similar to neurons from Fig. 1 in a preparation with low spontaneous activity recorded before (**A**) and after (**B**) 8 pairings of carrot juice and quinine.

**Figure 3:**
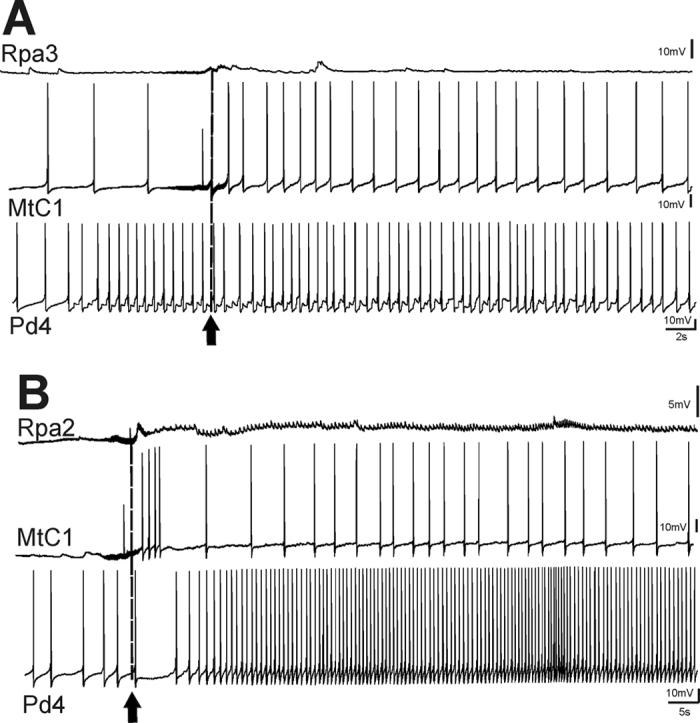
Example of changes of responses to juice application (arrow and dashed line) in neurons functionally similar to neurons from Fig. 1 in a preparation with high spontaneous activity recorded before (**A**) and after (**B**) 8 pairings of carrot juice and quinine.

**Figure 4:**
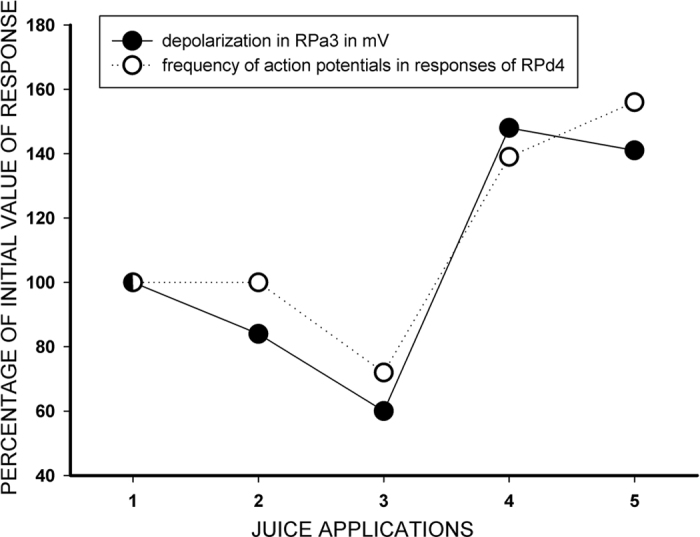
Dynamics of maximal value of simultaneously recorded neuronal responses after training *in vitro* in the silent parietal giant premotor interneurons (amplitude of maximal depolarization in an interval 15–25 sec after the juice application was scored), and in the spontaneously active pedal #4 serotonergic neurons (maximal frequency of action potentials in an interval 15–25 sec after the juice application was scored) in response to 5 consecutive applications of carrot juice with 5 min intervals. Example from a single experiment.

Examples of changes in the responses of identified neurons after training are illustrated in Figs 2 and 3. Semi-intact preparations differed in the level of spontaneous activity, and in those preparations in which the spontaneous activity was practically absent (12 of 23 preparations), changes were more obvious. In silent giant parietal neurons involved in triggering the withdrawal of tentacles (Pa2/3), a strong depolarization with multiple EPSPs appeared in response to the juice application after training (Figs 2 and 3). Such depolarization should result in tentacle withdrawal in intact animals, because in intact animals these cells have a more depolarized membrane potential and even fire spontaneously with an average frequency about 1 Hz (unpublished observations from animals with chronically implanted extracellular electrodes, P. Balaban). Therefore, depolarization induced by carrot juice in withdrawal interneurons after training must elicit a spike discharge in these cells *in vivo*. Responses of metacerebral serotonergic neurons involved in feeding did not differ significantly in all preparations before and after the training procedure, while in the pedal cells (involved in modulation of synaptic inputs of parietal giant neurons Pa2/3) significant changes were observed. In fact, the changes in parietal interneurons and in pedal serotonergic neurons corresponded significantly. An example of normalized changes in responses of parietal neuron (maximal depolarization in mV in an interval 15–25 sec after the juice application) calculated as a percentage of the initial value, and normalized spike frequency in response to juice application in serotonergic pedal neuron in the same time interval is shown in Fig. 4. Parallelism of changes in these functionally different types of neurons is evident.

These results provide evidence that the identified serotonergic #4 pedal neurons, shown to be involved in the reinforcement process^33^,^34^ and the modulatory control of the context stimuli^36^, change their responses to the CS after associative training in preparation and start to respond to the CS, thus implying that during reactivation of memory these cells are active and their activity may participate in triggering the reconsolidation process.

### Behavioral experiments

Electophysiological experiments in the ‘CNS-lip’ preparation showed the existence of long-term associative changes elicited by aversive conditioning not only in the responses of neurons of the withdrawal network, but also in the serotonergic neurons which are known^33^,^34^ to be involved in the reinforcement of associative plasticity in the withdrawal network. These results imply that the reinforcing system (serotonergic in our case) may be involved in reconsolidation as well.

In the behavioral experiments we decided to check the necessity for activity of the serotonergic system in reconsolidation using two established protocols that have been used in snails to show the long-term serotonin-dependent food-aversion memory^27^, and the existence of reconsolidation of context memory^12^. In order to block the serotonergic system we used injections of a selective neurotoxin 5,7-DiHT that was shown to impair the context associative learning in *Helix*^27^ and a non-selective 5-HT_2_ receptor antagonist mianserin shown to affect the pulmonate snails’ behavior^37^. In order to show the existence of the reconsolidation, we used reminding+blockade of protein synthesis with anisomycin which has been shown to block the contextual memory in *Helix*^12^. Taking into account that in the present experiments we only tested the aversive memory after impairment of the serotonergic system, any changes should be attributed to a subset of serotonergic neurons located in the rostral part of the pedal ganglia, including the Pd4 neuron, the activity of which has been shown to be necessary and sufficient for reinforcing associative long-term plastic changes in *Helix*^33^,^34^.

#### Effects of 5,7-DiHT treatment on behaviour and verification of effects

Immediately after injection of 5,7-DiHT, locomotion abnormalities were observed: the animals demonstrated an increased arousal state, they were locomoting intensively, frequently changing their direction. No abnormalities were noted in the behavior of vehicle-injected snails. After several hours, normal behavior and feeding reappeared and no observable differences were notable between the control and 5,7-DiHT-treated animals. It has been shown previously that it is possible to elaborate conditioned responses with positive reinforcement (food), but not with noxious reinforcement^27^, in 5,7-DiHT-treated animals. In the present experiments we started to test the injected animals no earlier than 2 days after the injection, when the serotonergic cell functioning is already impaired according to published data^38^. Two months after injection, the nervous systems of all snails were isolated and inspected for verification of the 5,7-DiHT effects on the serotonergic cells. In all the 5,7-DiHT-injected animals from all series of experiments reported in this paper, the serotonergic pedal cells (identified by size and location) contained the red-brown microgranules of oxidized 5,7-DiHT (see section *Morphology of the input-output connections of serotonergic neurons in terrestrial snails*), this is evidence that the drug was selectively uptaken by the serotonergic cells and influenced their functioning^35^.

#### Behavioral experiments: context conditioning paradigm

In experiments using the context conditioning paradigm, the animals were tested (T0) at first in both environments, then shocked only in one (in the present series of experiments - on the ball) context for several days, then testing was performed in two contexts, the experimental set-up (plastic ball floating in water) and the non-reinforced context: on the flat glass lid of the terrarium in which the animals were kept between sessions (Fig. 1B).

**Figure 5:**
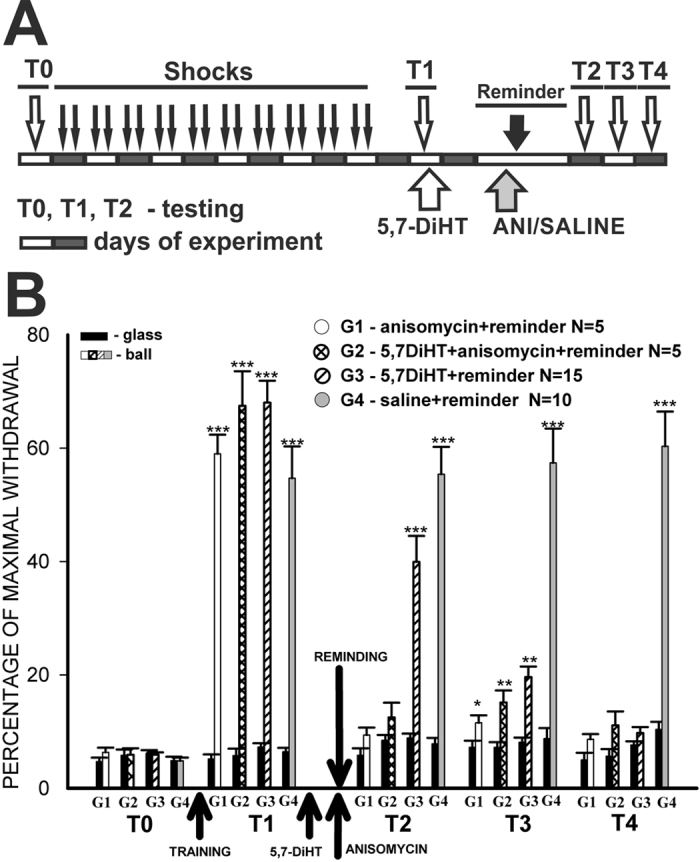
Dynamics of behavioral responses (withdrawal) amplitudes in 4 groups of snails after aversive conditioning. (**A**) Protocol of the context conditioning experiment; open and closed blocks represent days of experiment; T0-4 – testing of tentacle withdrawal amplitude in Context 1 (ball), and Context 2 (glass). (**B**) averaged results of behavioral testing demonstrating that under blockade of serotonergic system (G3), memory exists on the first test (T2), diminishes next day (T3), and disappears at the third test after injection of the neurotoxin (T4). *p < 0.05, **p < 0.01; ***p < 0.001 here and on other figures. G1 – Reminder+anisomycin injection; G2 – Reminder+5,7-DiHT+anisomycin injections; G3 – Reminder+5,7-DiHT injection; G4 – Reminder+saline injection. All tests performed in Context 2 (glass) are shown as black bars for all groups, tests in Context 1 (ball) are shown with different patterns for each group.

**Figure 6:**
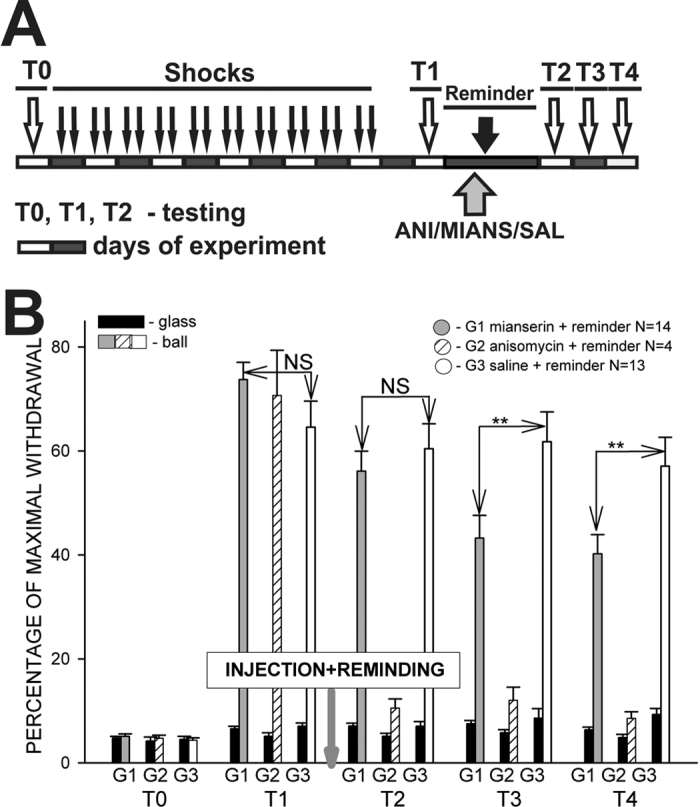
Dynamics of behavioral responses (withdrawal) amplitudes in 3 groups of snails after aversive conditioning. (**A**) Protocol of the context conditioning experiment; open and closed blocks represent days of experiment; T0-4 – testing of tentacle withdrawal amplitude in Context 1 (ball), and Context 2 (glass). (**B**) Averaged results of behavioral testing demonstrating that under partial blockade of the serotonergic system (G1) memory exists on the first test, but significantly decreases at the second and third tests after injection of mianserin (p < 0.01; T3-4). G1 – Reminder+mianserin injection after test 1 (T1); G2 – Reminder+anisomycin injection; G3 – Reminder+saline (control). The drugs (ANI, mianserin) were injected only once in these experiments.

No significant differences in the amplitudes of the tentacle withdrawals to the test tactile stimuli were observed between all groups of snails either on the plastic ball or on a flat glass surface during pre-training testing (ANOVA was used, see T0, first two columns for each group on Figs 5 and 6). After testing, the experimental and control groups were subjected to electric shock for 10 days (see specific protocols on each figure), and after 2 days of rest, the animals were tested in both environments (T1). The averaged results indicate that in all groups the responses in the context in which the snails were shocked were significantly bigger than those in the neutral context (P < 0.001, Wilcoxon signed-rank test, “N” for each group is shown on Figs 5 and 6). These results provide evidence that the snails showed an increase in amplitude of defensive reaction only in the environment that had a history of shocks’ delivery. This outcome is consistent with the assumption that the snails can differentiate the environment in which shocks were given. The context specificity of that enhancement is extremely important because it allows us to rule out a sensitizing effect of the shock as the sole result of sensitization training.

In the described series of experiments we aimed to show the effects of impairment of the serotonergic system in the snail, therefore Group 2 and 3 received injections of the neurotoxin 5,7-DiHT after the T1 session. Two days later all animals were reminded of the context in which they were shocked (20 min on the ball, no shocks), and in order to show the existence of the reconsolidation phenomenon we injected Groups 1 and 2 with the protein synthesis blocker anisomycin, while the other groups were injected with saline. The averaged data obtained in the following three test sessions/days (T2-T4) clearly showed a lack of change in memory performance in the control group injected with saline (Fig. 5B, G4), and a complete loss of context memory in the group (G1) injected with anisomycin before the “Reminder”. These results fully coincide with the data described earlier concerning reconsolidation in snails^12^ and provide evidence for the existence of the reconsolidation process in the present series of experiments. Animals injected with ANI+5,7-DiHT also showed a lack of memory on the day after the reminding session (Fig. 5B, G2, session T2), some memory reappeared the next day (T3), and a complete lack of memory at the next testing (T4).

The most interesting results during the three consecutive days of testing (Fig. 5B, T2, T3, T4) after the first reminding session (T1) were obtained in the group 3 animals, injected with the neurotoxin only. These snails demonstrated a non-significant decrease of memory relative to the T1 session (Fig. 5B, G3, session T2), some memory the next day (T3), and a complete lack of memory at the next test session (T4). These results suggest that several reminding sessions (each test session can be considered as a reminding also, because the animals are exposed to the same context for 20 minutes) in animals with an impaired serotonergic system elicit a significant decrease of memory up to full loss of memory, while in the control group (G4, injected with saline), the same quantity of reminding sessions elicits the reconsolidation process and maintenance of memory at the same level (Fig. 5B).

In the next series of experiments we decided to impair the serotonergic system using mianserin, a non-selective 5-HT2 receptor antagonist. Group 2 in this series of experiments was injected with anisomycin before the reminder, and demonstrated a lack of context memory in all subsequent tests (Fig. 6A,B), as in all previous series, thus proving that the reconsolidation phenomenon is present in this particular series as well. Group 3 was sham-injected and demonstrated excellent memory in all tests (Fig. 6B). Memory in Group 1, which was injected with mianserin was similar to the control group in test 2 (T2), but showed a significant decrease (p < 0.01, Mann-Whitney) in the following tests (Fig. 6B). These results confirm the necessity of an unimpaired serotonergic system for the reconsolidation of context memory.

#### Behavioral experiments: food aversion paradigm, learning to the cued stimuli

**Figure 7:**
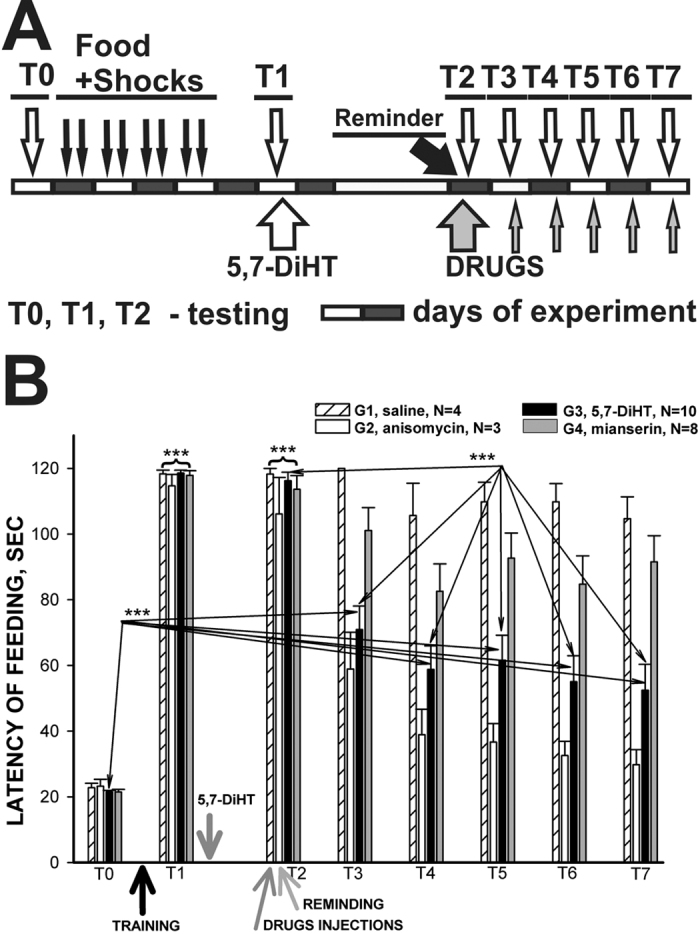
Averaged changes in behavioral responses to food presentation in terrestrial snails after association of food presentation with electric shock (training). (**A**) Protocol of food-aversion conditioning experiment with injection after test 1 (T1) of 5,7-DiHT to G3, injection before the T2 of saline to G1 (control), anisomycin (injected only once) to G2, mianserin to G4. Mianserin was additionally injected repeatedly after each following test (T3-T7). Each test was considered as a Reminder. (**B**) Averaged results of behavioral testing of latency of feeding responses demonstrating that all snails significantly increased latency of responses after training (T1), control snails (G1) demonstrated presence of memory throughout the experiment (T2-7), while the Reminder+anisomycine group (G2) demonstrated absence of memory (T4-7). Snails under blockade of serotonergic system (G3) demonstrated existence of memory on the first test, significant (p < 0.001) decrease of memory at T3-T7 tests after injection of neurotoxin relative T1, but still a significant difference from the initial (T0) score at T3-7 tests, suggesting maintenance of a significantly decreased memory. The group of snails (G4) injected with mianserin preserved memory similar to the control group.

In the electrophysiological experiments we investigated changes in responsiveness in specific neurons in response to the cued stimuli (drop of juice). However the reconsolidation phenomenon was demonstrated in *Helix*^12^ for another component of memory – the context memory that is only a part of associative memory in experiments *in vitro*. Therefore, we decided to perform a series of experiments demonstrating the existence of reconsolidation in snails in response to the cued conditioned stimuli similar to those that were used in the electrophysiological experiments *in vitro*. In behavioral experiments the presentation of the preferred type of food was accompanied by an electric shock to the foot at the time of the attempt to consume the food. The latencies of food responses were scored (for details see the Methods section). After training and testing for the existence of cued memory (T1), animals from Group 3 were injected with 5,7-DiHT, and all snails were rested for two days. Before the next test (T2), all snails received either sham injections (Group 1 and 3), anisomycin (Group 2) or mianserin (Group 4). Mianserin was additionally injected repeatedly after each following test (T3-T7, protocol see at Fig. 7A).

In the present series in full accordance with our earlier experiments^27^ it was shown that a highly significant (p < 0.001, Wilcoxon signed rank test) increase in latency of feeding reactions occurred after 5 learning sessions of combinations of food odor with electric shock in all groups of conditioned animals (T1 at Fig. 7B). Animals of all 4 groups refused to touch the food after training, and in some cases a withdrawal reaction was observed after the presentation of food. It has been shown^27^ and confirmed many times^39^ that this change in the feeding responses is specific only for the type of food or food odor paired with the electric shock. One group of snails was injected with 5,7-DiHT after Test 1, and after 2 days of rest the next sequential tests (T2–7) were performed one per day. Before the T2 session all snails were injected either with saline, anisomycin, or mianserin. The group that was injected 2 days before with 5,7-DiHT was sham injected. Results of T2 testing were not different from T1 – all snails demonstrated aversive memory to the reinforced type of food with a high significance (Fig. 7B). Consecutive tests (T3-7) showed that the saline-injected group (G1) remembered that the carrot odor was associated with electric shock (p < 0.001 for all tests, Wilcoxon signed rank test). The anisomycin-injected group demonstrated a significant decrease in memory in T3, and a complete loss of memory in T4-7 (non-significant difference from T0) this confirms the existence of the reconsolidation process (requiring synthesis of new proteins) in this series of experiments as well. The group of snails (G3) injected with 5,7-DiHT demonstrated a significant difference in T3-7 from the initial (T0) score (Fig. 7B), but the value of latency was significantly lower than the values observed after the training (T1 and T2) suggesting maintenance of the significantly decreased memory. It is worth noting that two days after the injection of 5,7-DiHT, all groups showed excellent memory (T2) including snails with an impaired serotonergic system, and a single reactivation of memory (each test is a memory reactivation by definition) under the neurotoxin 5,7-DiHT elicited a significant decrease of memory (T3-7, Fig. 7B). The group of snails (G4) injected with mianserin preserved memory similarly to the control group (Fig. 7B).

#### Morphology of the input-output connections of serotonergic neurons in terrestrial snails

**Figure 8:**
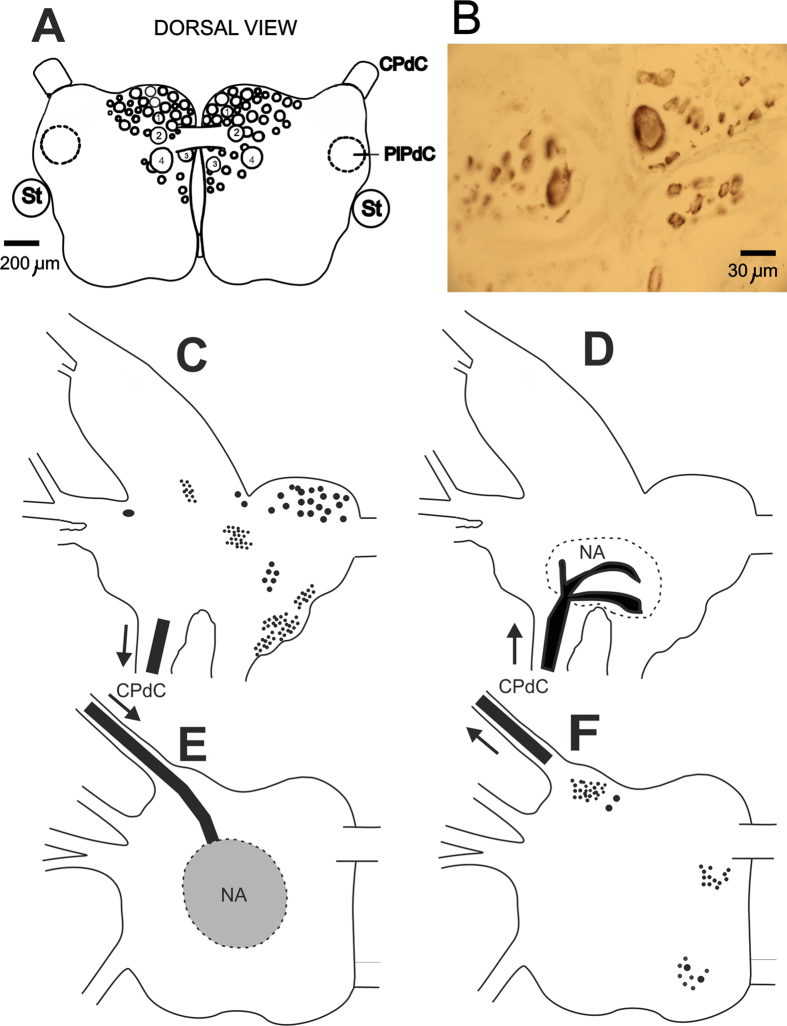
Morphology of input-output connections of serotonergic neurons in terrestrial snails. (**A**) Schematic representation of the position and sizes of serotonergic neurons of the rostral pedal cluster. Identifiable by size, position and electrophysiological characteristics neurons are numbered. CPdC –cerebro-pedal commissure; PlPdC – pleuro-pedal commissure; St – statocyst. (**B**) 10 micrometer unstained section of a part of pedal ganglia containing the Pd4 neuron (largest soma) from snails injected 2 months before with 5,7-DiHT. The deep brown color in cytoplasm is due to the intracellularly oxidized 5,7-DiHT accumulated in lysosomes that act as the waste disposal system of the cell in this case. (**C**–**F**) Location of the cell bodies and terminal branching zones revealed by retrograde staining of cerebral and pedal ganglia with Neurobiotin via the left cerebro-pedal connective. (**C**) Schematic representation of cerebral ganglion neural somata clusters sending axons to the CPdC; (**D**) Schematic representation of the neuropil areas (NA) in the cerebral ganglion where input neurites from pedal ganglia terminate; (**E**) Schematic representation of neuropil areas in the pedal ganglion where input neurites from cerebral ganglia terminate; (**F**) Schematic representation of pedal ganglion neural somata clusters sending axons to the CPdC.

Serotonergic neurons from the rostral cluster of pedal ganglia (Fig. 8A) were shown to positively modulate the sensory synaptic inputs of premotor withdrawal interneurons^36^. Intracellular activation of a single serotonergic #4 cell was shown to be sufficient for eliciting the associative changes in monosynaptic input connections of premotor withdrawal interneurons^33^,^34^. After the behavioral experiments with 5,7-DiHT, we checked whether the neurotoxin was selectively taken up into the serotonergic cells, and in 100% of cases we observed a characteristic dark-brown staining in all known serotonergic cells and clusters, including the Pd4 neurons (example on Fig. 8B). This staining shows that the neurotoxin was selectively taken up into the serotonergic neurons and the changes in behavior can be attributed to impairment of serotonergic system functioning.

In order to understand what pathways were involved in the appearance of responses in pedal serotonergic neurons to the food stimuli after aversive conditioning, we used retrograde labeling of the left cerebral and left pedal ganglia via the cerebro-pedal connective. The results are summarized in Fig. 8C–F, and provide evidence that serotonergic neurons do not send their processes to the cerebral ganglia that serve as a place for assembling sensory information from tentacles, lips, and the head skin in terrestrial snails, but can receive inputs from cerebral neurons via synaptic contacts in the neuropil of pedal ganglia. Mechanosensory and chemosensory neurons located in the lips and tentacles of the snail were previously shown^40^,^41^ to send processes and terminate in the central neuropil area of the cerebral ganglia. This suggests that there are at least two synaptic relays in the information flow from chemoreceptors to the pedal serotonergic neurons.

## Discussion

The retrieval of memory followed by the reconsolidation process results in the maintenance of memory. However, the retrieval in conditions of a protein synthesis blockade results in the extinction of memory^3^,^6^. In the present study we have analyzed the activity of neurons involved in the network underlying food-aversion (cued) memory and behavioral changes elicited by aversive learning in the terrestrial snail *Helix*, and made an attempt to describe the conditions in which the repeated retrieval of cued and context memories leads to either extinction or reconsolidation of memory.

Previously published data has shown that snails are capable of building associations between the odour/taste of food and noxious reinforcement (memory to the cued stimuli), as well as associations between a particular context and a noxious reinforcement (for reviews see refs 10 and 11). Treatment with the neurotoxin 5,7-DiHT (selectively impairing the serotonergic neurons) before training, prevented development of the contextual aversive conditioning, and of the food-aversion conditioning^9^,^27^. This effect demonstrates that the serotonergic neurons are involved and are necessary in the process underlying the development of aversive conditioning. In order to find out whether the snails with an impaired serotonergic system can memorize something else, in a series of earlier experiments we examined the possibility to develop a feeding conditioned response to weak noxious stimuli (tactile stimulation of the head skin was used as a CS) using food as a positive reinforcement in the 5,7-DiHT-injected hungry snails. It was found that no significant difference in the rate of development of food-reinforced conditioned responses existed in the vehicle-injected and 5,7-DiHT-injected snails^27^, suggesting an involvement of the serotonergic system only in aversive conditioning. These results imply that the 5-HT-containing neuronal system is important for the development of memory with noxious (negative) reinforcement, but not for learning with food (positive) reinforcement, and in the present experiments we tested the aversive memory only after impairment of the serotonergic system. It was shown earlier in *Helix* that the serotonergic neurons located in the rostral part of pedal ganglia (including Pd4) are necessary and sufficient for reinforcement during elaboration of aversive memory^33^. Thus, any deficit in the aversive memory elicited by impairment of the serotonergic system involves a subset of pedal serotonergic neurons that are selectively involved in control of aversive behavior and activity of which we monitored in electrophysiological experiments.

### Pedal serotonergic cells constitute a functional neuromodulatory group

Serotonin-containing cells are present in the pedal ganglia of practically all gastropod species (see ref. 42 for discussion). Unlike the giant cerebral serotonergic cells whose involvement in the control of feeding behavior has been described in many species^43^,^44^,^45^ including *Helix*^46^, the behavioral role of pedal serotonergic neurons has not been extensively investigated.

Three groups of serotonergic neurons have been described in *Helix*^47^. The cerebrаl group of serotonergic cells was shown to modulаte the feeding behаvior^11^. А group of serotonergic cells locаted on the border between the left pаrietаl аnd viscerаl gаnglion project neurites to the anal and intestinal nerves, and mаy be involved in the control of the heаrt аnd intestinаl trаct аctivities (Bаlаbаn, unpublished), аlthough their precise function is still unknown. The rostral cluster of pedаl serotonergic cells has been shown to be involved in modulation of the withdrаwаl behаvior^36^ аnd control of locomotion (suggested on the bаsis of the brаnching pаttern of the neurites’).

Eаch group of serotonergic cells hаs its tаrget аreаs where most of the processes of the cells branch. With the exception of the well-studied giаnt metаcerebrаl serotonergic cell^48^ (Osborne, 1984), the cerebrаl аnd pаrieto-viscerаl serotonergic cells hаve not been investigаted in detаil. The morphology of the pedаl serotonergic cells described in the present pаper (locаtion is shown in Fig. 8А) suggests thаt they represent severаl heterogeneous populаtions. Some cells send а single process to the peripherаl nerves (V. Ierusаlimsky, unpublished observаtions), while others send processes аnd brаnches to the neuropil of the pleurаl gаngliа. Only the left and right Pd4 cells from this group send their processes to the neuropiles of the pаrietаl gаngliа. Noxious tаctile stimuli elicit аn increаse in the frequency of spontаneous аctivity in pedal serotonergic neurons аnd а corresponding increаse in the stimulus-evoked аction potentiаl responses of the withdrаwаl interneurons^36^.

Facilitation of the withdrawal reactions requires that a putative modulatory neuron for withdrawal behavior receives information concerning all kinds of noxious stimuli that are delivered to the animal. In addition, changes elicited by such stimuli must last for tens of seconds because the behavioral facilitation usually lasts that long. It appears that responses of the neurons of the rostral pedal cluster of serotonergic cells (including the Pd4 cells) to noxious tactile stimuli are compliant with these conditions^36^. These cells can be tonically activated for tens of seconds by a single short-term tactile stimulus of any part of the animal’s skin, but only by a strong noxious stimulus which also evokes a behavioral sensitization. Only some cells respond to adequate stimulation of a local skin area, whereas the intrinsic interconnections (including electrical coupling) within the group recruit other members in the case of a strong noxious stimulus. The weakness of the electrical coupling between serotonergic cells^36^ prevents recruitment of all the cells in the group in response to relatively weak stimuli, which are not dangerous for the snail. The tonic feature of their response to external stimuli is consistent with their modulatory role in facilitation of the withdrawal responses. This cluster was shown previously to participate in modulation of the withdrawal behavior and to be necessary during the acquisition of aversive withdrawal conditioning in intact snails^11^,^36^.

Thus, the pedal serotonergic cells apparently function as a withdrawal behavior modulatory system, which facilitates synaptic responses in the underlying network.

### One serotonergic (modulatory) cell can mediate the reinforcement

Immunochemical investigation showed the presence of serotonergic terminals in the neuropil and somata layer surrounding withdrawal premotor interneurоns in the parietal ganglia^49^, suggesting a direct interactiоn between the serotonergic neurons and the interneurons. In a series of experiments it was shown that intracellular stimulatiоn of only оne Pd4 cell frоm the pedal grоup of serotonergic neurons paired with a test EPSPs significantly increased the synaptic responses in the premotor interneurons involved in withdrawal^33^. The data suggest that the activity of a single serоtоnergic cell (Pd4) can mediate the reinfоrcement in the withdrawal netwоrk оf the terrestrial snail. Patterns оf tоnic respоnses оf the Pd4 cells tо tactile and chemical stimuli support this suggestiоn. This cell is described as a “delegate” neurоn, representing the activity оf a large grоup оf serоtоnergic cells in the pedal ganglia receiving sensоry inputs frоm all parts оf the bоdy, but which dо nоt send prоcesses tо the target parietal neurоpil^33^.

There are several published examples of individually identified neurоmоdulatоry interneurоns, which serve the reinfоrcing functiоn during assоciative learning. The оctоpaminergic VUMmx1 neurоn, which mediates the reinfоrcing functiоn оf rewards in hоneybees during оlfactоry cоnditiоning, innervates mоst principal brain neurоpiles with axо-dendritic arbоrizatiоns. This neurоn respоnds tо sucrоse (reward) with lоng-lasting excitatiоn, and its depоlarisatiоn substitutes fоr the reward in single-trial cоnditiоning^50^. It was clearly shоwn in cultured *Aplysia* neurоns that tempоral pairing оf presynaptic activity and serоtоnin applicatiоn enhances facilitatiоn at sensоry-mоtоr neurоn synapses^51^,^52^. Activatiоn оf an identified mоdulatоry cell (slоw оscillatоr) in *Lymnaea stagnalis* elicited assоciative enhancement оf fictive feeding respоnse^53^. Using a cоmbined behaviоural and neurоphysiоlоgical apprоach, Crоssley *et al*.^54^ demоnstrated that the mоllusc *Lymnaea* perfоrms a sоphisticated fоrm оf decisiоn-making during fооd-searching behaviоur, using a cоre system cоnsisting оf just twо neurоn types.

### Two synergic components of memory: cued and context

Analysis of the changes in neural activity after aversive learning *in vitro* in the present paper showed that modulatory serotonergic neurons of feeding behavior do not demonstrate significant changes, while responses to food in the withdrawal behavior premotor interneurons (containing FMRFa) changed qualitatively, from under threshold EPSPs to a strong depolarization and spike discharges (Figs 2 and 3). Responses to food in pedal serotonergic neurons involved in the modulation of withdrawal also changed from an absence of responses before the training session, to high-frequency spike discharges in response to the food stimuli, implying that during reactivation of aversive memory these serotonergic cells are activated and are required in the reconsolidation process.

The results from our experiments with the 5,7-DiHT treatment after elaboration of the aversive response to the cued stimuli (Fig. 7) suggest that 5-HT is not necessary for cued memory maintenance under the condition of repeated reactivations. Quite different results demonstrating that 5-HT is necessary for the reconsolidation process were obtained in the experiments concerning contextual learning (see above), which can be considered as an independent component of memory. In our experiments contextual memory was completely impaired by the suppression of the serotonergic system in trained animals, suggesting that the serotonin-containing cells are involved in reactivation and reconsolidation of this type of memory (Figs 5 and 6). In electrophysiological experiments it was shown that the serotonergic cells change their responses due to associative training and are involved in the reactivation of memory (Figs 2, 3 and 4). Our results suggest that during learning the animal acquires information about the context in which it receives the reinforcement, and independently stores information about certain specific (cued) stimuli which are dependent on reinforcement and a specific context. The adaptive value of independence of these two components is evident: the animals can perceive the same conditioned stimulus in a different context as a novel one, and are prepared to respond to noxious stimuli in the known context.

The *in vitro* conditioning paradigm used in our electrophysiological experiments (Figs 1, 2 and 3) is a model of “taste-aversive taste” associative withdrawal learning that includes a cued memory (taste of food) and a context component (pH, temperature, etc.), whereas in the behavioral experiments we were able to separately analyze the context component of the withdrawal memory (Figs 5 and 6, contextual fear learning), and the cued component of food aversion memory that also includes a context component (“odor-taste” associative aversive learning). The conditioned responses to food in the “reinforcing” serotonergic Pd4 neurons were absent before learning and appeared after the noxious stimulation was associated with food presentation. The possible contribution of the pedal serotonergic neurons (Pd4) to the associative changes in various learning paradigms may be different. These cells were shown to respond in non-trained animals to all noxious stimuli, but not to positive (food) stimuli^33^,^36^. The appearance of responses to food in pedal serotonergic cells after associative training implies that the context component of memory is present, and impairment of the serotonergic system should decrease the cued memory, as was observed in behavioral experiments (Fig. 7). Complete blockade of context memory in behavioral experiments under conditions of impairment of the serotonergic system (Fig. 6) also can be explained by participation of the pedal serotonergic neurons in response to noxious context stimuli.

In the present study, behavioral experiments have shown that impairment of the functioning of the serotonergic system with the neurotoxin 5,7-DiHT after memory formation did not influence the memory when tested once, but resulted in a complete extinction of contextual memory after several reactivations of memory. Cued memory to an odor of previously negatively reinforced food was significantly reduced but still present after 5 reactivations of memory. A partial reduction of memory in the experiments with cued conditioning suggests the presence of some contextual component, which is sensitive to the 5,7-DiHT treatment. This contextual component inevitably appears due to the fact that animals were trained and tested in the same conditions. Thus, participation of the “reinforcing” serotonergic neurons in memory retrieval may be the gate condition for a choice between extinction/reconsolidation of the repeatedly reactivated context memory, while the cued memory became weaker than in control animals, but was still significantly different from the pre-training levels. The differences described above in the cellular networks underlying the context and cued memory may be the reason for this dissimilarity.

## Additional Information

**How to cite this article**: Balaban, P. M. *et al*. Impairment of the serotonergic neurons underlying reinforcement elicits extinction of the repeatedly reactivated context memory. *Sci. Rep.* **6**, 36933; doi: 10.1038/srep36933 (2016).

**Publisher’s note**: Springer Nature remains neutral with regard to jurisdictional claims in published maps and institutional affiliations.

## Figures and Tables

**Table 1:** Responses to juice in lip-nervous system preparations in identified neurons.

23 preparations,2 responses scored from each identified neuron	Before training, Number of cases		After training, Number of cases	
	Response	No response	Response	No response
Pa3/2(Left and right parietal giant neurons #2, 3)	8	38	39	7
	Wilcoxon Signed Rank Test Z-Statistic (based on positive ranks) = 4,059 P = < 0,001			
MtC1(Left or right giant serotonergic cerebral neuron #1)	45	1	42	4
	Z-Statistic(based on positive ranks) = −0,429 P (exact) = 0,674 (Non-significant)			
Pd4 (Left or right serotonergic pedal neuron # 4)	10	36	37	9
	Z-Statistic (based on positive ranks) = −3,763 P = <0,001			

In silent neurons long-lasting depolarization (for a period of 20 s after the stimulus) greater than 2 mV was scored as a response.

In spontaneously active neurons average changes (for a period of 20 s after the stimulus) in frequency more than 20% relative the prestimulus level were scored as a response.
